# Tourist Landscape Preferences in a Historic Block Based on Spatiotemporal Big Data—A Case Study of Fuzhou, China

**DOI:** 10.3390/ijerph20010083

**Published:** 2022-12-21

**Authors:** Fan Liu, Danmei Sun, Yanqin Zhang, Shaoping Hong, Minhua Wang, Jianwen Dong, Chen Yan, Qin Yang

**Affiliations:** 1 College of Landscape Architecture and Art, Fujian Agriculture and Forestry University, Fuzhou 350100, China; 2 Engineering Research Center for Forest Park of National Forestry and Grassland Administration, Fuzhou 350002, China

**Keywords:** landscape preference, big data, heat map, historic blocks, landscape planning

## Abstract

Historic blocks are valuable architectural and landscape heritage, and it is important to explore the distribution characteristics of tourists to historic blocks and their landscape preferences to realize the scientific construction and conservation of historic blocks and promote their sustainable development. At present, few studies combine the analysis of tourist distribution characteristics with landscape preferences. This study takes the historic block of Three Lanes and Seven Alleys in Fuzhou as an example, combines field research and questionnaires to construct a landscape preference evaluation indicator system for the historic block, measures the distribution characteristics of tourists in the block through the heat value of tourist flow obtained from the Tencent regional heat map, and analyses the influence of landscape preference indicators on the heat value of tourist flow in the block through stepwise multiple linear regression. The research shows that: (1) the spatial and temporal variation in the heat value of tourist flow tends to be consistent throughout the block, from 7 a.m. to 6 p.m., showing a “rising, slightly fluctuating and then stabilizing” state, both on weekdays and on weekends. (2) The factors influencing the heat value of tourist flow in the different spatial samples are various, with commercial atmosphere, plant landscape, accessibility of the road space, architecture, and the surrounding environment having a significant impact on the heat value of tourist flow. Based on the analysis of the landscape preferences of tourists in the historic block, a landscape optimization strategy is proposed to provide a reference for the management and construction of the block.

## 1. Introduction

In the public realm, no element is more important than the block, an active place to work, shop, dine out and engage in daily activities [1]. Blocks have evolved along with the city and are a significant part of it, with a variety of types, spatial forms, and increasingly complex functions. According to the relevant research directions, current studies on blocks are multifaceted and may be divided into studies on physical and mental health [2,3,4], perceived safety [5,6], audio-visual quality [7,8], thermal comfort [9], traffic safety [10], economics and ethnicity [11,12], street crime [13], etc. Tourists always tend to choose attractive blocks for leisure and recreation, and the historic block is the most important material and cultural heritage in the city, carrying the memory of a city’s development, highlighting the characteristics of the city, giving tourists a unique recreational experience, with great potential and value for development [14]. However, while attracting a large number of tourists, the historic block is often marked by the phenomenon of overcrowding, causing congestion in popular streets and alleys, which places a burden on the block environment and affects the visiting experience. At the same time, in the process of accelerating urban construction, as the urban development cycle shortens, the space for landscape and traditional cultural activities in historic blocks is being compressed by the rapidly developing modern economy, and historical blocks generally exhibit the phenomenon of serious commercialization and serious homogeneity of block renovation. The construction of historic blocks is biased towards the scale of urban development at the expense of core cultural, spiritual, and aesthetic values, and ignores public aesthetic preferences and local cultural contexts, which is not conducive to the sustainable development of the blocks.

With the demand for urban transformation and urban renewal in China, the shaping of the landscape of historic blocks to serve the aesthetic perception and cultural spirit of the urban public has become a major issue that needs to be addressed. In 2014, UNESCO pointed out at the International Symposium on the “Historic Urban Landscape” that landscape is a new type of inheritance management method. Moreover, it is of great significance to study the development and construction of historic blocks from the perspective of landscape [15,16,17]. The landscape of the historic blocks contains visible material forms of space (such as structures and the natural environment), but also has a non-material sense of spiritual and cultural connotations, and is the basis for the condensation of the characteristics of the regional tourism image. The purpose of preserving and improving the landscape of the historic blocks is to meet the multi-dimensional needs of the district in terms of cultural conservation [18], promoting landscape tourism [19], and improving the quality of life of the community [20]. The landscape of historic blocks is the carrier of an important part of their value, and tourists’ landscape preference for historic blocks has become an important basis for block construction. Landscape preference is a concept that involves both the biophysical characteristics of the natural environment and the subjective perception of humans. Previous studies have typically used questionnaires and field surveys to collect public evaluation of landscape preferences, which requires a considerable amount of human and material resources [21,22,23,24]. Recently, with the development and popularization of big data, there are several more scientific, efficient, and objective research methods of landscape preference, which can make space protection and landscape construction develop in a better direction. For instance, Tieskens et al. (2018) proposed a robust big data methodology using social media photos from Flickr and Panoramio to estimate the correlation between landscape attributes and landscape preferences, in order to pinpoint what it is in a landscape that attracts people. The method successfully linked the structural elements of the landscape with the revealed preferences, providing a way of quantifying the appreciation of the landscape [25]. Dong et al. (2022) used heat value obtained from Baidu heat maps and spatial big data, such as points of interest, Open Street Map and online review data, to analyze the factors affecting the vitality of urban green spaces from two dimensions, the internal and external spatial characteristics of green spaces, and propose corresponding optimization suggestions [26]. Liu et al. (2022) used open GPS-trajectory data, Markov chains, and cluster analysis to analyze the spatiotemporal patterns of tourists’ movement behaviors in Mount Huashan in China, so as to come up with a strategy for the spatial optimization of the scenic area [27]. Ding et al. (2022) conducted a comparative evaluation of 45 mountain landscapes in Beijing based on social media data. Big data capture, semantic network analysis, and importance–performance analysis were used to explore the composition of tourist groups in mountain parks, the preferences of the tourist groups, and the relationships between park tourists and different influencing factors, and to evaluate the recreational experiences of tourist groups [28]. Kong et al. (2022) used big data techniques to obtain 55,441 web-based textual data on urban park evaluations to quantify the emotional states of visitors, and used Random Forest to identify urban park attributes that were correlated with positive emotions. This sheds new light on the relationship between park landscape patterns and visitors’ positive emotions [29]. These studies use advanced big data technology to make data more accessible and sample sizes larger, while the findings are more scientific and reliable, providing some assistance in urban planning and ecological construction.

The heat map is a visual representation of the density of points in a map through a density function, and it is displayed on the map by means of different colors, brightness overlays, etc. It is used to reflect the distribution of people in the area and the trend of change, which can reflect the preferences of tourists in the area to a certain extent [26,30,31,32]. The data includes the latitude and longitude of the collection points as well as the flow of people, from a wide range of sources, with accurate data and good real-time performance [33]. Therefore, we decided to use the heat values obtained through big data techniques as an external representation of tourists’ preferences, combined with the tourist landscape preference data obtained from questionnaires, to explore the landscape preference indicators affecting tourist distribution and to try to build a linear regression model, adopting a “statistical data + network big data” fusion approach. We thus break through the traditional static model based on statistical data and the limitations of the relevant data, using a large volume of real, multi-source, network stream data, and multi-dimensional data fusion of innovative research methods to verify and complement each other. Thus, this study attempts to combine the heat value of tourist flow from a heat map with the landscape preferences of tourists to carry out research, while the Three Lanes and Seven Alleys in Fuzhou, China is selected as the object of this study. The study sought to determine:

1. A landscape preference evaluation system for tourists visiting Three Lanes and Seven Alleys;
2. Patterns of the spatial and temporal distribution of tourists and landscape preference characteristics in Three Lanes and Seven Alleys;
3. The relationship between the heat value of tourist flow and landscape preferences, and an attempt to construct a linear regression model.

The results of the study will help the future landscape space planning and construction of the blocks to develop in a direction that will lead to tourist satisfaction, and ultimately provide reference advice on the concrete implementation of the landscape and landscape management construction in Three Lanes and Seven Alleys.

## 2. Materials and Methods

### 2.1. Study Area

**Figure 1:**
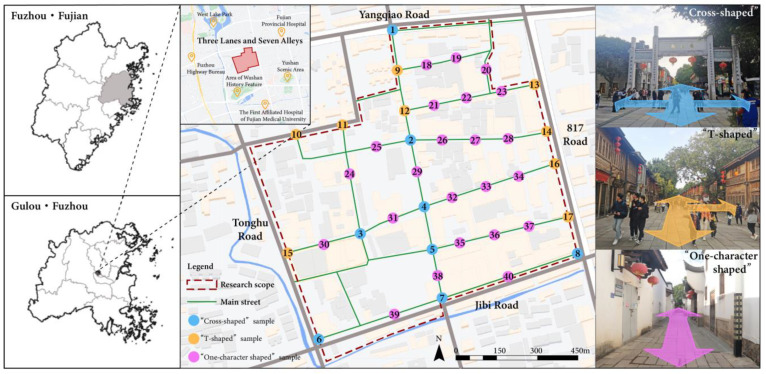
The geographical location of the Three Lanes and Seven Alleys and the specific location of the study sample site. The blue dots represent the “Cross-shaped” spatial samples, the yellow dots represent the “T-shaped” spatial samples, and the purple dots represent the “One-character shaped” spatial samples.

The survey was conducted in Fuzhou city. Fuzhou is located on the southeast coast of China, in the east of Fujian Province, and is the capital of Fujian Province. Fuzhou is a famous historical and cultural city, and Three Lanes and Seven Alleys is the quintessential core of the history and culture of this ancient city. Three Lanes and Seven Alleys, consisting of three squares, seven lanes, and a central street, is the most representative historic block in Fuzhou, an important cultural root and symbol of the city. Historic blocks are blocks with a high concentration of cultural relics and monuments, or where the traditional style and national and local characteristics of a certain historical period can be more completely reflected. The Three Lanes and Seven Alleys basically retains the unique pattern of lanes and alleys left over from the Tang and Song dynasties, and the architectural style of the Ming and Qing dynasties, and the private gardens in the block have the characteristics of traditional Fuzhou gardens. A total of 270 ancient residences exist, and there are 131 historically protected ancient buildings. With the development of the city, The Three Lanes and Seven Alleys has been transformed from a residential area to a historic block consisting of historical buildings, museums, exhibition halls, shops, etc. In 2010, The Three Lanes and Seven Alleys was named a national 5A tourist attraction and in 2012, it was selected as one of China’s World Cultural Heritage sites. With reference to the scope of the conservation planning for the historic block of Three Lanes and Seven Alleys, the scope of the study was defined through field surveys and research: the study area follows the surrounding roads, from the east to 817 Road, from the west to Tonghu Road, from the north to Yangqiao Road, and from the south to Jibi Road, with a total land area of about 34 hm^2^ (See Figure 1).

In Kevin Lynch’s *Urban Imagery*, the five elements of paths, boundaries, areas, nodes, and signs are mentioned, of which the nodal space is one of the important elements in the study of urban imagery, with the role of connection, aggregation, and symbolism. In historic blocks, nodal spaces are generally located at the intersection of the streets and in areas within the streets that have a landscape and cultural character. The spatial form of the nodes in Three Lanes and Seven Alleys is presented in different states, from the plane, which is “Cross-shaped” to the “T-shaped” formed by the intersection of streets, and the “One-character shaped” inside the lanes. According to the spatial characteristics of the nodes, 40 node samples were selected in the intersecting paths of Three Lanes and Seven Alleys, including 8“Cross-shaped” spatial samples, 9 “T-shaped” spatial samples and 23“One-character shaped” spatial samples, as shown in Figure 1.

### 2.2. Building a Landscape Preference System for Tourists and Analysis of Landscape Preference Evaluation

This study is based on grey statistic analysis (GSA), used to screen the landscape preference indicators of tourists visiting Three Lanes and Seven Alleys. The use of GSA to screen indicators can avoid compromises in the findings or the influence of extreme outliers due to differences in expert opinions, and improve the objectivity and scientific validity of indicator selection [34].

#### 2.2.1. Initial Collection of Evaluation Indicators

In terms of landscape preference research, a large number of indicators have been proposed in previous studies. On this basis, through a thorough understanding of the characteristics of Three Lanes and Seven Alleys, literature review, theoretical analysis, and field survey, combined with consultation with experts in landscape architecture and related fields, a collection of 24 evaluation indicators of landscape preference of tourists to Three Lanes and Seven Alleys was initially formed, which can be generalized into three large classes of landscape environment dimension, spatial dimension, and social and humanistic dimension.

#### 2.2.2. Survey on the Degree of Importance of Evaluation Indicators

Firstly, we created a questionnaire based on the preliminary selection of landscape preference indicators, and in order to ensure the scientific nature of the questionnaire and data analysis, and not to give psychological hints to the respondents, the 24 indicators were randomly arranged. Then, a questionnaire on the importance of the landscape indicators of Three Lanes and Seven Alleys was distributed to 30 experts in the relevant research fields, and 30 valid questionnaires were returned. The evaluation scale is based on a seven-point Likert scale, and Cronbach’s α function in SPSS 25.0 (IBM, Chicago, America)was used to check the reliability of the data.

#### 2.2.3. Building Grey Class Whitening Functions

Using grey statistical analysis, we constructed segmentation functions for the pre-selected indicators of landscape preference according to three levels: ‘high’, ‘medium’, and ‘low’. Define the function f_k_(ij) to denote the value of the whitening function for the jth indicator with the importance degree of i rank, where i = 1, 2, 3,…, 7; j = 1, 2, 3,…, 24; k is the number of grey classes, and assign the value of k = 1, 2, 3; h_ij_ is the value of the jth pre-selected indicator with the importance degree of i rank [35]. The f_k_(ij) segmentation function is calculated by the formula:

Whitening function with ‘high’ importance, k = 1

$$f_{1}\left(\mathrm{ij}\right)=\{\begin{array}{c} 1\;h_{\mathrm{ij}}\geq 7\\ \frac{h_{\mathrm{ij}}-4}{7-4}\;4<h_{\mathrm{ij}}<7\\ 0\;h_{\mathrm{ij}}\leq 4 \end{array}$$

Whitening function of ‘medium’ importance, k = 2

$$f_{2}\left(\mathrm{ij}\right)=\{\begin{array}{c} 0\;h_{\mathrm{ij}}\geq 7\\ \frac{7-h_{\mathrm{ij}}}{7-4}\;4<h_{\mathrm{ij}}<7\\ 1\;h_{\mathrm{ij}}=4\\ \frac{h_{\mathrm{ij}}-1}{4-1}\;1<h_{\mathrm{ij}}<4\\ 0\;h_{\mathrm{ij}}\leq 1 \end{array}$$

Whitening function of ‘low’ importance, k = 3

$$f_{3}\left(\mathrm{ij}\right)=\{\begin{array}{c} 0\;h_{\mathrm{ij}}\geq 4\\ \frac{4-h_{\mathrm{ij}}}{4-1}\;1<h_{\mathrm{ij}}<4\\ 1\;h_{\mathrm{ij}}\leq 1 \end{array}$$

#### 2.2.4. Calculate Grey Class Decision Coefficients and Compare Grey Class Decision Vectors

The grey class decision coefficient is the basis of the grey decision vector. The grey class decision vector for each evaluation indicator consists of three levels of “high”, “medium”, and “low”. Through statistical analysis of the returned questionnaire data, three grey class decision coefficients of η_high_, η_medium_, and η_ow_ were calculated for each pre-selected evaluation indicator [36]. The formula is as follows:

$$\eta_{k}\left(j\right)=\sum \;L\left(\mathrm{ij}\right)\;\times \;f_{k}\left(\mathrm{ij}\right)$$

where (j) is the decision coefficient of the jth evaluation indicator belonging to the kth grey class; L(ij) is the number of experts who score the jth pre-selected indicator with importance degree i; f_k_(ij) is the value of the whitening function for the jth evaluation indicator with importance degree i.

By comparing the three decision coefficients in the grey decision vector, the maximum value of the vector will determine the importance of the selected evaluation indicator, and only those indicators with “high” importance will be filtered out, thus finally determining the evaluation indicators of the landscape preference of tourists to Three Lanes and Seven Alleys.

#### 2.2.5. Evaluation of the Landscape Preferences of Tourists to Three Lanes and Seven Alleys

In order to study the influence of each landscape indicator on tourists’ landscape preferences, photographs were taken of the 40 selected sample areas, taking full account of the landscape information contained in each photograph, and a questionnaire was developed in conjunction with the identified landscape preference indicators. The results were collated and analyzed to obtain the quantitative values of each landscape indicator for each area through an on-site questionnaire survey in which 120 tourists scored the actual conditions of each sample site with scores of [−2,2], thus making an evaluation of tourists’ landscape preferences.

### 2.3. Methods for Evaluating the Heat Value of Tourist Flow

#### 2.3.1. Data Acquisition

**Figure 2:**
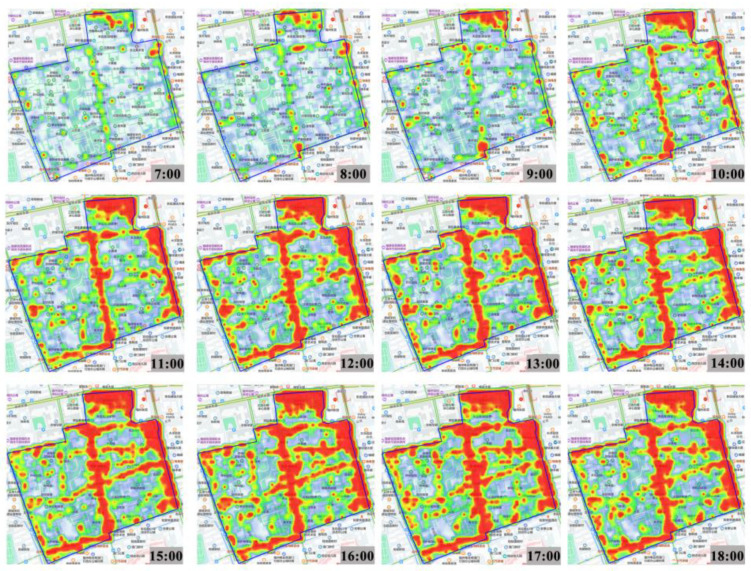
Map of heat value of tourist flow (part).

Tencent regional heat map is a big data visualization product that effectively reflects the spatial distribution of phenomena and geographical events in the relevant region, generating data information in real time within the region. This part of the data comes from the location data of the Three Lanes and Seven Alleys scenic area obtained from the Tencent regional heat map. Taking into account the impact of the opening hours of museums and exhibition halls on the flow of tourists in Three Lanes and Seven Alleys, the impact of major holidays on the flow of tourists, and the fact that the data obtained avoid extreme weather conditions such as rain, fog, and extremely high or low temperatures, the location data from 11 October 2022 to 16 October 2022, from 7 a.m.–6 p.m. every day was selected as the study data. The data were collected at regular intervals of one hour during this period of time, from which the data were selected for the three days of 11 October, 12 October, and 13 October on weekdays and 15 and 16 October on weekends as samples, and a total of 60 heat maps were obtained (see Figure 2). According to the location of the sample areas and the corresponding width of each street, the heat information within approximately 50 m^2^ of each sample area was intercepted as the heat value of tourist flow, with a total of 120,000 samples. With a visual accuracy of 100 m and data granularity down to the individual, the Tencent regional heat map can be used as a reflection of the flow of people in each area, indicating tourists’ landscape preferences to some extent.

#### 2.3.2. Data Processing

In this study, the heat map of tourist flow is assigned a heat value (See Table 1) from 1 to 7, respectively, based on the seven color change characteristics of the heat map (i.e., purple, blue, cyan, green, yellow, orange, and red). The closer the color is to red, the higher the relative population density, and the closer it is to purple, the lower the population density [37].

We pre-processed the data by means of data format conversion, etc., gridded the data, formed the same color areas into faceted shapes, combined with the Three Lanes and Seven Alleys road drawings, and imported them into CAD for area calculation, thus quantifying the intercepted heat map, obtaining the heat value data of the tourist flow of each place at each time period, and superimposing the individual layer data for summation [26,33]. The total heat value of tourist flow within each sample area is derived from Equation (1), the formula being as follows:

(1) $$V=\sum_{i=1}^{7}S_{i}\times i$$

where V is the total heat value of tourist flow, i is the heat value of tourist flow of the area corresponding to the heat map, and S_i_ is the area of the region corresponding to i.

The above method was used to obtain the heat value of tourist flow for each time period within each area, and the average daily heat value of tourist flow from 7 a.m. to 6 p.m. within a day was calculated separately for each sample site according to Equation (2) [26,33]. The formula is as follows:

(2) $$H=\frac{\sum^{\;}V_{t}}{T}$$

where H is the mean heat value of tourist flow in the sample area, T is the time period counted, and V_t_ is the total heat value of tourist flow in the sample area at different time periods.

Using thermal analysis, we can quantify the sample areas where tourists congregate to understand the pattern of tourist distribution in Three Lanes and Seven Alleys.

### 2.4. Building a Linear Regression Model

This study hypothesizes that there is a linear relationship between the heat value of tourist flow at each area in Three Lanes and Seven Alleys and the landscape indicators, with the aim of exploring the influence of the landscape indicators on the heat value of tourist flow. We first conducted a correlation analysis using SPSS25.0(IBM, Chicago, America) to compare the quantified evaluation indicators with the aim of verifying their correlation. Subsequently, we used multiple linear regression to eliminate indicators with low or no significant correlation impact for analysis and identify the key indicators that affect the heat value of tourist flow [38,39]. The formula is as follows:

$$Y=a\;+\;b_{1}\;\times \;X_{1}\;+\;b_{2}\;\times \;X_{2}\;+\;\ldots \;+\;b_{n}\;\times \;X_{n}$$

where Y is the dependent variable, a is a constant term, X_1_, X_2_,…X_n_ are the independent variables, and b_1,_ b_2_…b_n_ are the undetermined parameters.

During the modeling process, the adjusted R^2^ was used to test the validity of the model, and the variance inflation factor (VIF) was used to check for multicollinearity and avoid outliers in the model [40,41].

## 3. Result

### 3.1. Landscape Preference System Construction and Evaluation Analysis

#### 3.1.1. Landscape Preference System Construction

A questionnaire on the degree of importance of the landscape indicators of Three Lanes and Seven Alleys was distributed to 30 experts in the relevant research fields, (10 professionals engaged in research related to the construction of the historic block and 20 professors of landscape architecture, architecture, and urban and rural planning,) and 30 valid questionnaires were returned. Based on the experts’ scores, the decision coefficients of each pre-selected indicator were calculated, 16 evaluation indicators with “high” importance were screened out, and 8 evaluation indicators with “medium” importance were excluded. The results of the specific statistical analysis are shown in Table 2. Thus, the evaluation system of the landscape preferences of tourists in Three Lanes and Seven Alleys was finally constructed (See Table 3).

#### 3.1.2. Analysis of the Results of the Evaluation of the Landscape Indicators

**Figure 3:**
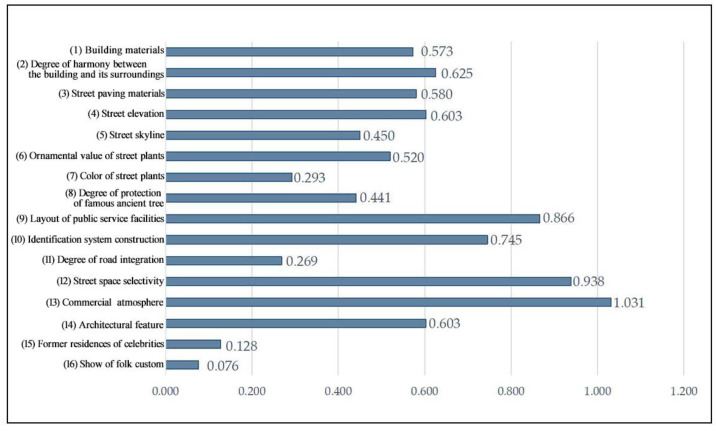
Evaluation values of landscape preference indicators for the “Cross-shaped” spatial sample.

All landscape preference indicators were positive in the “Cross-shaped” spatial sample (see Figure 3). The highest score of 1.031 was obtained for (13) commercial atmosphere, indicating a high degree of commercial development and a strong commercial atmosphere in the “Cross-shaped” spaces, which have increased economic benefits based on conservation and regeneration. However, the lowest scores were obtained for (16) show of folk custom and (15) former residences of celebrities, with scores of 0.076 and 0.128 respectively, indicating that folklore culture and local activities are not reflected in the environment in the “Cross-shaped” sample area, so that folk customs and culture can be integrated into the landscape in future planning and construction. Moreover, the “Cross-shaped” spatial sample is concentrated at the intersection of the streets and alleys, where there are fewer former residences of celebrities, and therefore scores low for the corresponding indicator.

**Figure 4:**
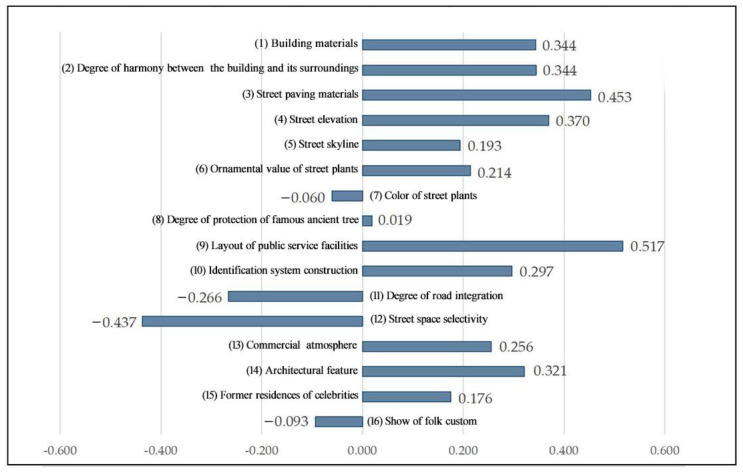
Evaluation values of landscape preference indicators for the “T-shaped” spatial sample.

In the “T-shaped” spatial sample, the highest score of 0.52 was given to the (9) layout of public service facilities indicator (see Figure 4). This indicated that public service facilities in this type of space are relatively well built and systematic, providing convenience for tourists while integrating the historical and local characteristics of Three Lanes and Seven Alleys into the design in terms of style and appearance, and giving tourists a good sensory experience by harmonizing with the environment in the space. Among these indicators, the three indicators with the lowest scores were, in descending order, (12) street space selectivity (−0.437), (11) degree of road integration (−0.266), and (16) show of folk custom (−0.093). The analysis shows that the reasons for the low scores for selectivity and integration are similar, as some of the “T-shaped” spatial samples are on the periphery of the whole block and are less accessible to the whole block, which lowers the average score. This is a common phenomenon in the development of tourist attractions, as commercial development in the block is a hindrance to the development of traditional folk culture, which is not sufficiently promoted.

**Figure 5:**
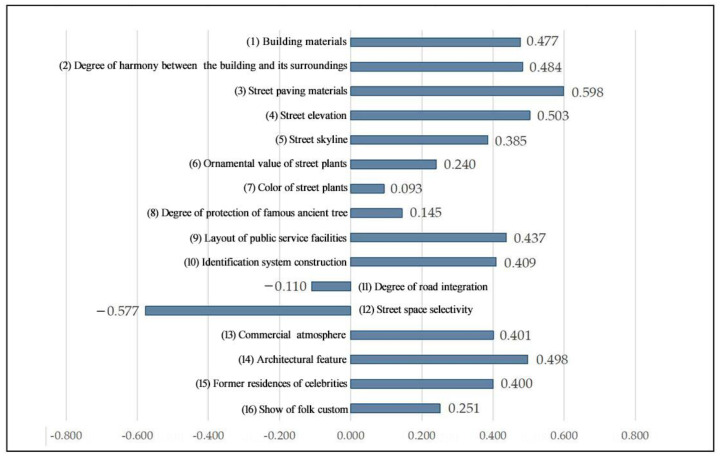
Evaluation values of landscape preference indicators for the “One-character shaped” spatial sample.

As can be seen from Figure 5, the three highest-scoring landscape preference indicators are, in descending order, (3) street paving materials (0.598), (14) architectural features (0.498), and (4) street elevation (0.503), indicating a strong historical atmosphere in the overall space of the street and lane, the uniformity and local characteristics of the landscape of the street and lane, as well as the preservation of the remaining structures and the dimensional pattern of the streets and lanes in the spatial sample. The three lowest-scoring landscape preference indicators were (12) street space selectivity (−0.577), (11) degree of road integration (−0.110), and (7) color of street plants (0.093). This showed that the “One-character shaped” spatial sample is less accessible and has a lower potential for gathering tourists than the other two. In the future, the spatial optimization of the block can be used to improve the ability to attract tourists to the area, with a view to reasonably dispersing the flow of tourists along the main street. In addition, there are fewer colorful plant landscapes with few plants in the streets, so the score of this indicator is also low.

### 3.2. Analysis Results of Heat Value of Tourist Flow

**Figure 6:**
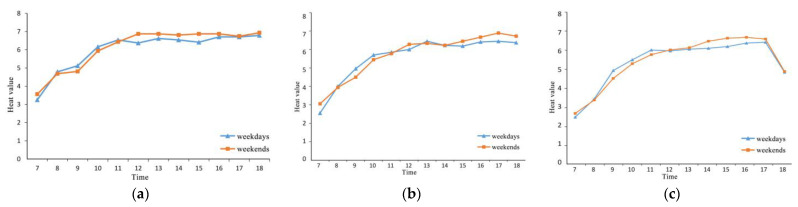
Variation in heat value of tourist flow between weekdays and weekends for the three spatial samples: (**a**) for the “Cross-shaped” spatial sample, (**b**) for the “T-shaped” spatial sample, and (**c**) for the 1 “One-character shaped” spatial sample.

Through the heat value of tourist flow from the heat map for weekdays and weekends, we obtained the results of the spatial and temporal distribution of heat values for different types of sample areas. As can be seen in Figure 6, the heat value of tourist flow for weekdays is generally higher than for weekends in three types of sample areas before 12 a.m., while after 12 a.m. the heat value of tourist flow for weekends will exceed that for weekdays. At the same time, from the changes in the heat value of tourist flow on weekdays and weekends from 7 a.m. to 6 p.m., it is found that there is a certain regularity in the change in the heat value of tourist flow in Three Lanes and Seven Alleys, and the heat value of tourist flow in different spatial sample area as a whole goes through a process from increasing to gradually stabilizing. From 6 a.m. to 12 a.m., the heat values of the various spatial sample areas show a gradual increase; they reach a peak around 12 a.m. and tend to plateau or decline slightly, before reaching a new peak between 3 p.m. and 5 p.m. It is worth noting that only the “One-character shaped” sample starts to show a significant decrease in heat value after 5 p.m. This is probably due to the fact that the exhibition halls and featured buildings in the “One-character shaped” spatial sample are one of the main tourist attractions in the area. They generally close at 5 p.m., which may be the main reason for the longitudinal decrease in tourist flow after 5 p.m. in this type of area. The other two types of areas maintain a high level of tourist flow mainly due to their relatively high accessibility and the abundance of commercial facilities.

According to Table 4, for the “Cross-shaped” spatial sample area, sample area 1 has the highest heat value of tourist flow (6.256) on weekdays, because sample area 1, as the main north entrance of Three Lanes and Seven Alleys, has an open space, which is conducive to the gathering and dispersal of tourist flow. Furthermore, this area has a spectacular, historical, and iconic gatehouse marked ‘South Back Street’, where tourists often gather to take photographs. The highest heat value of tourist flow during weekends is in sample area 7, with a heat value of 6.346. This sample area is the main entrance/exit to the south of Three Lanes and Seven Alleys and has a strong commercial atmosphere and the ability to gather tourist flow. The lowest ranking in terms of the heat value of tourists on both weekdays and weekends is sample area 3, indicating that this area is less attractive to tourists, which may be related to its remote location away from the main street.

In the “T-shaped” spatial sample, the highest heat value of tourist flow on weekdays and weekends was for sample area 16, as it is an entrance to the interior of Three Lanes and Seven Alleys from 817 Road, and there is a resting green space in the sample, which has a good effect of planting landscape and is conducive to the stay and gathering of tourists. The lowest heat values of tourist flow on weekdays and weekends were found in sample area 9 and sample area 11, respectively, which, when combined with the field survey, is due to the lack of attractive landscape elements in these two sample areas and the fact that tourists are less likely to visit them.

In the “One-character shaped” spatial sample, the highest heat value of tourist flow on weekdays was for sample area 20, which connects Three Lanes and Seven Alleys with the Dongbai commercial area, increasing the aggregation of pedestrian flow to a certain extent. The highest heat value on weekends was for sample area 22 with a score of 6.08. In this sample space, the plant colors are bright and prominent, and the skyline is in harmony with the picture. Then, the lowest heat value of tourist flow on weekdays was for sample area 37, which is located deeper in the lane, with lower accessibility and fewer landscape elements in the sample space to attract tourists to the area, making it less capable of gathering tourist flow. Meanwhile, the lowest score in the heat value for tourist flow on weekends was for sample area 40, which is located on a peripheral road, with poor accessibility and a lack of colorful planting, making it less attractive to tourists. Therefore, in the “One-character shaped” space of the lane, the sample area with high accessibility and strong colorful plants in the block will help attract tourists to the area.

### 3.3. Regression Analysis between the Heat Value of Tourist Flow and Landscape Preference

This study hypothesizes that there is a linear relationship between the heat values of tourist flow and landscape preference indicators for the three spatial samples of Three Lanes and Seven Alleys. Firstly, the data from each of the three spatial sample areas were collated and correlation analysis was carried out for 16 landscape preference indicators. Subsequently, multiple linear regression analysis was carried out and 32 stepwise regression models (7 models for the “Cross-shaped” spatial sample, 8 for the “T-shaped” ones, and 17 for the “One-character shaped” ones) were obtained by gradually eliminating uncorrelated influencing factors through stepwise regression models. By comparing the model fit value of R^2^ and the adjusted R^2^, the results of ANOVA analysis, and the VIF values, the optimal models were finally selected for each of the three sample areas (See Table 5).

From Table 5, we can learn that the model has a good fit in the “Cross-shaped” spatial sample (R^2^ = 0.515, adjusted R^2^ = 0.434 > 0.03, and F = 6.367). After a multiple stepwise regression, we get an indicator: (13) commercial atmosphere, with a significance of 0.045 < 0.05, indicating that it is a landscape preference indicator that significantly affects the heat value of tourist flow. If the VIF values are less than 10, there is no covariance problem; therefore, the model of the “Cross-shaped” spatial sample can be constructed as follows:

Y_1_ = 4.519 + 1.181X_13_

where Y_1_ is the value of tourist flow in the “Cross-shaped” spatial sample, 4.519 is a constant term, and X_13_ is the commercial atmosphere.

In the “T-shaped spatial” sample, the R^2^ and adjusted R^2^ were 0.922 and 0.882, respectively, showing a good degree of model fit and also passing the F test. After multiple stepwise regression, seven indicators were obtained, of which the (7) color of street plants and (10) identification system construction had *p*-values greater than 0.05 and were excluded. The remaining five indicators, on the other hand, all significantly affect the heat value of tourist flow, while their VIF values are all less than 10, so that the model of the “T-shaped” spatial sample can be constructed as follows:

Y_2_ = 6.685 + 1.939X_15_ − 2.052X_13_ + 3.829X_8_ − 5.015X_2_ + 2.424X_6_

where Y_2_ is the value of tourist flow in the “T-shaped” spatial sample, 6.685 is a constant term, X_15_ is the former residences of celebrities, X_13_ is the commercial atmosphere, X_8_ is the degree of protection of famous ancient trees, X_2_ is the degree of harmony between the building and its surroundings, X_6_ is the ornamental value of street plants.

The model has a good fit (R^2^ = 0.742, adjusted R^2^ = 0.433 > 0.4, and F = 3.580) in the “One-character shaped” spatial sample; after multiple stepwise regression, five indicators are obtained, of which the influence of (14) architectural features (*p* = 0.15 > 0.05) is weak and has been removed. The VIF values for the remaining four landscape preference indicators were all less than 10, and the model did not suffer from covariance. Therefore, the model of the “One-character shaped” spatial sample can be constructed as follows:

Y_3_ = 4.524 + 3.009X_11_ + 0.803X_7_ − 0.695X_12_ + 1.533X_5_

where Y_3_ is the value of tourist flow in the “One-character shaped” spatial sample, 4.524 is a constant term, X_11_ is the degree of road integration, X_7_ is color of street plants, X_12_ is street space selectivity, and X_5_ is the street skyline.

## 4. Discussion

Historic blocks distill the character and connotations of a city and are the areas with the most prominent urban landscape features. The popularity of big data has improved the accessibility of the data and the ability to process and calculate the data, improving the reliability and scientific validity of the data samples and the analysis results, which provides new ideas and methods to carry out research related to historic blocks. Here, we conducted a study combining public aesthetics and the heat value of tourist flow for landscape preferences.

### 4.1. Introducing the Tencent Heat Map to Measure the Spatial and Temporal Distribution of Tourists

The use of historic blocks reflects the degree of support and development of the blocks for human life. Moreover, people are the mainstay of urban development and the driving force behind the development and preservation of historic blocks [42]. The use of heat maps in previous studies has generally focused on spatial vitality and has not yet been correlated with tourist landscape preferences [43,44]. Therefore, we chose the Tencent heat map, a spatial type of big data that directly reflects the temporal aggregation of people, as an external representation of tourists’ landscape preferences. Due to the ease, speed, and real-time nature of the visualization features of the big data heat map, the crowd density, the speed of footfall, and the heat of clustering in each area can be quickly calculated for each period clustered within the open space, resulting in a spatial distribution of the heat values of tourist flow for different place types. Through data analysis, we obtained the ranking of heat values for each type of spatial sample and also found that the distribution of tourists on the streets of Three Lanes and Seven Alleys changed significantly over time. Therefore, the study indicates that there is a certain pattern in the spatial and temporal distribution of tourists in the recreational areas of the Three Lanes and Seven Alleys historic block and that there is a correlation between the characteristics of tourists’ activities and landscape preferences, which confirms the views of Liu (2022) et al. [27] and Shao (2017) et al. [45]. The historic block attracts tourists because of its unique landscape, and the landscape and the functions it contains play a certain role in guiding the behavioral activities of tourists, who gather in some spaces of the block according to their different recreational needs. Meanwhile, the landscape environment of the block also influences tourist behavior and the degree of convergence, so the use of big data such as heat maps to capture the spatial and temporal distribution of tourists and to explore the characteristics of the landscape environment in different areas has positive relevance, as has been demonstrated in previous studies. For example, Wang et al. (2012) also point out that understanding the temporal and spatial distribution of tourist behavior can provide information on tourists’ recreational needs, which can then be planned and updated accordingly to actively promote the development of scenic areas [46].

Nowadays, with the rapid development of tourism, excessive tourism in historic blocks has become a key issue for the sustainable development of the blocks. The influx of tourists exceeding the carrying capacity of blocks can lead to congestion, long waiting times, injuries, or damage to the block, which ultimately undermines the tourist experience and is detrimental to the sustainable development of the historic block [47,48,49]. Therefore, we analyzed the spatial and temporal distribution of tourists in Three Lanes and Seven Alleys and obtained the spatial and temporal differences in the heat value of tourist flow for different types of street sample areas. Then, by analyzing the landscape information contained in each area, we can initially determine the influence of tourists’ landscape preference on the heat value of tourist flow in different types of sample areas. Based on the different spatial types of the blocks as delineated in a previous study, the landscape environment in each area can be optimized in a targeted manner in the future to enhance the overall landscape appearance of the block and optimize the spatial layout. At the same time, the management of the block can also take timely diversion measures according to the distribution characteristics of the tourist flow to ensure the safety, comfort, and enjoyment of the tourists’ recreational experience, which is of positive significance in achieving mutual coordination between the development and conservation of the block. Finally, this part of the study also provides the basis for the next multiple regression analysis.

### 4.2. Relationship between Heat Values of Tourist Flow and Landscape Preferences

This study used multivariate stepwise linear regression analysis to investigate the key factors influencing the heat values of tourist flow, and the identification of the influencing indicators in the model has a significant impact on the analysis of the heat values. The content and number of indicators should cover all aspects of landscape preference; otherwise, the analysis can lead to an unclear use of the heat value of tourist flow and be unable to identify a direction for optimization. Based on previous research, we combined our current research and expert questioning to identify indicators of landscape preference through the GSA method. Depending on the type of spatial sample, we obtained different results. These landscape preferences can be summarized in terms of their meaning: commercial atmosphere, plant landscape, accessibility of the road space, architecture, and the appearance of the surroundings.

For the “Cross-shaped” spatial sample, the commercial atmosphere is the only landscape preference indicator that significantly affects the heat value of tourist flow, with a positive correlation. This suggests that an appropriate enhancement of the commercial atmosphere of the sample area will increase the tourist gathering capacity of the area, which is consistent with Zhang et al. (2021) [50], but different from Wang et al. (2015) [51]. At the same time, the commercial atmosphere is also a significant influence on the heat value of tourist flow in the “T-shaped” spatial sample, but they are negatively correlated, probably because this type of sample is generally located at the intersection of the site with external roads, where tourists are usually just about to visit the historic block or have already finished their visit, and the commercialization of the block is not the main factor attracting them to this area, so they are less concerned about this indicator. It can be seen that the impact of a commercial atmosphere is not the same in all sample areas and that the commercialization of a historic block needs to be tailored to the specific situation. Excessive commercialization may destroy the original landscape appearance and spatial layout of the historic block, and the question of how to grasp the extent of commercial development deserves consideration in future research. The plant landscape has shown a positive and significant impact on the spatial sample of the Three Lanes and Seven Alleys historic block. Previous studies have also confirmed similar findings [50,52]. This indicates that in a block space with a heavy sense of historical atmosphere, the use of plant color changes and brightness changes to create landscape nodes can effectively attract and retain tourists, making the area a high heat value area, and good plant landscape can greatly enhance the tourists’ play experience. At the same time, accessibility of the road space, architecture, and the appearance of the surroundings are two significant elements that influence the heat value of tourist flow in the historic block, and these two landscape indicators largely influence the overall landscape and pattern of the block and its cultural heritage, which are also often the main factors that attract tourists to the block [53].

Therefore, in the future construction of the historic block, it is possible to focus on these aspects according to the different spatial samples and to carry out appropriate landscape enhancement for those spaces that do not have a strong ability to attract tourists, so as to optimize the overall landscape appearance of the block and to relieve the pressure on the high heat value areas. Determining the impact indicators for the heat value of tourist flow is a complex and large task that requires continuous elaboration and validation of the model indicators. The selection of impact indicators in this paper is mainly based on the perspective of landscape preference, and the selection of impact indicators only takes into account the block itself, without taking into account the impact of the block’s surrounding environment on it. Subsequent studies could add impact factors to this paper for a more comprehensive study.

### 4.3. Suggestions for Enhancing the Landscape of Historic Blocks

Based on the results of the study on the landscape preferences of tourists to Three Lanes and Seven Alleys, combined with the current characteristics of the block, and following the objective rules of its development, some recommendations are made:

Firstly, maintain the original pattern of the block. The street pattern has always been one of the most important factors affecting the built environment of the block, and we should maintain the integrity of the traditional landscape of the block, with a focus on the preservation of the remaining historical buildings, street scale features, paving material forms and famous ancient trees. In the future, a balance should be struck between the historical and cultural heritage and the spirit of development to present the most authentic aspects of the local historical and cultural features to tourists.

Secondly, shape important nodal spaces. We should shape the nodal spaces with cultural, landscape, and local characteristics in the block, shape cultural spaces next to historical monuments or cultural protection units, shape landscape nodes with diverse elements at the corners of streets and roads and in grey areas enclosed by buildings, and set up public spaces at the entrances and exits of the block or in open areas. The interaction of the three types of nodal spaces can enhance the guided tour of the block, improve the feeling of the tourists’ experience, and increase the vitality of the block.

Thirdly, introduce commercial development properly. Planning the commercial layout rationally and focusing on increasing the proportion of commercial shops in the more sparsely populated squares and alleys can enhance the attractiveness of the area to a certain extent. In the main street and areas with good commercial development, the degree of commercialization should be grasped to avoid over-development and damage to the landscape appearance of the block. We can make use of characteristic elements such as the shape of traditional buildings and incorporate them into the design of shop facades to improve the unity between the commercial space of the block and the traditional historical space of the square.

Finally, improve the block service system and establish a feedback mechanism for tourists. The perfect conditions of service facilities will directly bring convenience to tourists and improve the quality of their visit. We should develop and improve the signage system, which can be combined with big data technology to create a smart block, where tourists can choose their own tour route and other services according to their needs. At the same time, tourists’ perceptions and the needs of the block should be taken into account and targeted for improvement by establishing feedback mechanisms, for example, through the judicious use of online evaluation data to determine what is hindering the use of the block.

### 4.4. Limitations and Implications

There are some limitations to this study. Firstly, the heat values of tourist flow we obtained are based on a web-based big data platform, and this part of the data will lack information on user attributes such as age, gender, place of residence, occupation, income level, and education level [54]. These can also be important indicators of tourists’ landscape preferences. At the same time, the heat map in the data acquisition mainly shows the scale and changing trend of the crowd, which does not reflect the subjective consciousness and behavioral purpose of the individual, and does not optimally represent the tourists’ touring pattern, which can be further optimized and supported by combining with other data sources in the future. It is important to emphasize that big data processing methods should be combined with other methods rather than being seen as a stand-alone approach. Future research may arrange for extensions or additions to address our current limitations.

Secondly, we have selected only one historic block, which is rich in commercial resources as well as old architectural resources, and the findings and recommendations are applicable to this type of historic block. In future research, we will consider a comparative study of historic blocks in multiple cities or select other types of historic blocks for study. In addition, seasonality is a characteristic of tourist mobility behavior. Future research may analyze the impact of seasons, months, and holidays on the spatial and temporal patterns of tourism activity.

## 5. Conclusions

In recent decades, as urbanization has accelerated, many cities have blindly planned and developed historic blocks in the pursuit of rapid development and economic benefits; meanwhile, the advent of the new data age has pushed planning and development in a more scientific and rigorous direction.

The landscape appearance of historic blocks is particularly important in planning and construction as the main form of presentation of their character and content. Based on domestic and international research, we selected the Three Lanes and Seven Alleys as the research object, and used big data analysis, questionnaires, and statistical analysis to understand the distribution pattern and landscape preference characteristics of tourists in the block, to explore the correlation between landscape preference indicators and the heat value of tourist flow in each area, and to summarize the key influencing factors that affect the heat value of tourist flow in the street space: commercial atmosphere, plant landscape, road space accessibility, architecture, and the appearance of the surroundings. We proposed directions and strategies for the optimization of future landscape construction of the block, which will serve as a reference for historic blocks in terms of landscape space enhancement.

## Figures and Tables

**Table 1:** The scores corresponding to the different colors of the heat map.

Color of Heat Map	Purple	Blue	Cyan	Green	Yellow	Orange	Red
**Value** **of Heat Map**	1	2	3	4	5	6	7

**Table 2:** The importance of landscape indicators of Three Lanes and Seven Alleys.

Type		Indicator	The Score of Importance			Level of Importance	Chosen or not
Large Class	Medium Class		“High” Level	“Medium” Level	“Low” Level		
Landscape environment dimension	Building	Building materials	17.015	13.667	0.333	High	Yes
		Degree of harmony between the building and its surroundings	22.383	8.625	0.000	High	Yes
	Street of block	Street paving materials	16.654	14.354	0.000	High	Yes
		Street elevation	21.365	9.314	0.334	High	Yes
		Street skyline	17.683	12.000	1.325	High	Yes
	Planting arrangement	Ornamental value of street plants	18.000	12.678	0.335	High	Yes
		Seasonal variation of street plants	12.345	16.677	1.993	Medium	No
Landscape environment dimension	Planting arrangement	Color of street plants	14.683	13.9954	2.338	High	Yes
		Degree of protection of famous ancient trees	21.022	9.324	0.663	High	Yes
	Landscape sketch	Sculpture	8.983	19.035	2.991	Medium	No
		Activity display	11.992	17.026	1.992	Medium	No
	Infrastructure	Distribution of parking lots	11.333	15.693	3.982	Medium	No
		Layout of public service facilities	15.000	14.674	1.333	High	Yes
		Identification system construction	15.985	14.036	0.996	High	Yes
Spatial dimension	Spatial indicator	Degree of road integration	19.372	11.33	0.332	High	Yes
		Clarity of road class	11.664	18.681	0.663	Medium	No
		Road connectivity	14.335	16.014	0.661	Medium	No
		Road visibility	12.366	16.653	1.993	Medium	No
		Street space selectivity	19.032	8.987	2.994	High	Yes
Social and humanistic dimension	Commerce	Commercial atmosphere	14.037	13.644	3.333	High	Yes
	History	Architectural feature	20.363	8.978	1.672	High	Yes
		Former residences of celebrities	18.344	11.993	0.673	High	Yes
	Folk custom	Show of folk custom	15.356	14.651	1.000	High	Yes
		Folk craft demonstrations	14.334	15.341	1.335	Medium	No

**Table 3:** Landscape preference indicator system.

Type		Indicator
Large Class	Medium Class	
Landscape environment dimension	Building	(1) Building materials
		(2) Degree of harmony between the building and its surroundings
	Street of block	(3) Street paving materials
		(4) Street elevation
		(5) Street skyline
	Planting arrangement	(6) Ornamental value of street plants
		(7) Color of street plants
		(8) Degree of protection of famous ancient trees
	Infrastructure	(9) Layout of public service facilities
		(10) Identification system construction
Spatial dimension	Spatial indicator	(11) Degree of road integration
		(12) Street space selectivity
Social and humanistic dimension	Commerce	(13) Commercial atmosphere
	History	(14) Architectural feature
		(15) Former residences of celebrities
	Folk custom	(16) Show of folk custom

**Table 4:** The heat value of tourist flow in different types of sample areas on weekdays and weekends.

Type of Sample Area	No.	Weekdays	Weekends
“Cross-shaped” spatial sample	1	6.256	6.154
	2	6.205	6.231
	3	3.590	4.308
	4	5.872	5.808
	5	5.795	5.731
	6	5.615	5.423
	7	6.154	6.346
	8	6.103	6.192
“T-shaped” spatial sample	9	4.436	4.923
	10	5.128	5.115
	11	4.513	4.231
	12	5.821	5.923
	13	4.949	4.962
	14	5.974	5.962
	15	4.821	5.038
	16	6.256	6.192
	17	6.051	5.923
“One-character shaped” spatial sample	18	5.026	4.500
	19	5.231	4.846
	20	6.051	5.731
	21	4.538	4.769
	22	5.641	6.077
	23	5.513	5.346
	24	4.282	4.077
	25	4.692	4.269
	26	4.410	4.885
	27	3.949	4.808
	28	4.154	4.846
	29	5.718	5.923
	30	4.513	3.962
	31	4.718	4.692
	32	3.872	4.885
“One-character shaped” spatial sample	33	4.744	4.731
	34	5.667	5.269
	35	3.974	3.923
	36	3.436	3.269
	37	3.128	3.462
	38	5.641	5.615
	39	5.564	5.538
	40	3.256	3.115

**Table 5:** Results of the multiple linear regression analysis.

Type of Sample Area		Unstandardized Coefficients		Standardized Coefficients	*p*	VIF	R^2^	Adjusted R^2^	F
		B	Standard Error	Beta					
“Cross-shaped” spatial sample	(Constant)	4.519	0.524		0.000				
	(13) Commercial atmosphere	1.181	0.468	0.718	0.045	1.000	0.515	0.434	6.367
“T-shaped” spatial sample	(Constant)	6.685	0.033		0.003		0.998	0.993	1109.628
	(15) Former residences of celebrities	1.939	0.039	2.516	0.013	2.102			
	(13) Commercial atmosphere	−2.052	0.031	−2.465	0.010	1.864			
	(8) Degree of protection of famous ancient trees	3.829	0.057	4.213	0.010	3.733			
	(2) Degree of harmony between the building and its surroundings	−5.015	0.151	−5.579	0.019	2.020			
	(6) Ornamental value of street plants	2.424	0.059	2.986	0.016	4.379			
	(7) Color of street plants	−0.459	0.040	−0.529	0.055	6.287			
	(10) Identification system construction	−0.161	0.047	−0.198	0.181	5.895			
“One-character shaped” spatial sample	(Constant)	4.524	0.327		0.000		0.742	0.433	3.580
	(11) Degree of road integration	3.009	0.963	1.275	0.006	5.81			
	(7) Color of street plants	0.803	0.355	0.464	0.037	1.47			
	(12) Street space selectivity	−0.695	0.327	−0.909	0.049	6.389			
	(5) Street skyline	1.533	0.651	2.354	0.031	4.189			
	(14) Architectural feature	−1.119	0.742	−0.597	0.15	5.454			

## Data Availability

The data used to support the findings of this study are available from the corresponding author upon request.
